# Identification of Most Stable Endogenous Control Genes for MicroRNA Quantification in the Developing Mouse Lung

**DOI:** 10.1371/journal.pone.0111855

**Published:** 2014-11-04

**Authors:** Wafae Bouhaddioui, Pierre R. Provost, Yves Tremblay

**Affiliations:** 1 Reproduction, Mother and Youth Health, Centre de recherche CHU de Québec, Québec, QC, Canada; 2 Department of Obstetrics/Gynecology & Reproduction, Faculty of Medicine, Université Laval, Québec, QC, Canada; 3 Centre de Recherche en Biologie de la Reproduction (CRBR), Faculté de Médecine, Université Laval, Québec, QC, Canada; Naval Research Laboratory, United States of America

## Abstract

MicroRNAs (miRNAs) are endogenous small non coding RNAs acting as negative regulators. miRNA are involved in lung development and pulmonary diseases. Measurement of their levels by qPCR is directly influenced by the stability of normalization gene(s), which can be affected by the experimental conditions. The developing lung is a changing tissue and one normalization gene showing stability on one developmental day may be modulated over time. Moreover, some developmental events are affected by sex, which also has to be considered. In this study, we compared stability of five putative control genes in the lung between sexes from the pseudoglandular to the alveolar stages and in adult lungs. Expression of *sno135*, *sno142*, *sno202*, *sno234*, and *sno251* was studied by qPCR in male and female lung samples collected at seven time points from GD 15.5 to PN 30. Cq values of *sno251* showed the highest variation across the different developmental stages, while *sno234* was the most stable gene. Gene expression stability was studied by geNorm, NormFinder and BestKeeper. Our data showed that ranking of genes based on expression stability changed according to developmental time and sex. *sno135*/*sno234* and *sno142*/*sno234* were proposed as best combinations of normalization genes when both sexes and all the studied developmental stages are considered. Normalization of let7-a RNA levels with different pairs of control genes proposed by geNorm and NormFinder gave similar data, while the use of less stable genes introduced a statistically significant difference on PN 0. In conclusion, variations in stability of normalization gene expression are observed over time and according to sex during lung development. Best pairs of normalization genes are presented for specific developmental stages, and for the period extending from the pseudoglandular to the alveolar stages. The use of normalization genes selected for their expression stability is essential in lung development studies.

## Introduction

MicroRNAs (miRNAs) are small non coding RNAs of ∼22 nucleotides [1]. They are endogenous regulatory molecules that negatively regulates gene expression [1]. The two first discovered miRNAs were reported in 1993 (Lin 4) and 2000 (let-7) [2],[3]. This class of molecules are involved in different physiological and pathological processes. miRNAs are highly conserved across plants, micro-organisms and animals [4]. The miRBase (online database of miRNA sequences [5], [6]) contains so far 2578 mature miRNA sequences and 1872 precursor human miRNA sequences, while 1908 and 1186 mature and precursor miRNA sequences are reported respectively for the mouse. It was shown that over 60% of human protein-coding genes are conserved targets of miRNAs [7]. The lung miRNA expression profile is highly conserved among mammalian species [8], [9]. miRNAs are involved in homeostasis and lung development [10], inflammation and viral infections [11], [12], and many pulmonary diseases such as cancer [13] and chronic obstructive pulmonary disease [14]. Knowledge on the role of miRNAs in lung development is still limited and based mainly on data from animal models.

Among quantification techniques used to study miRNAs, quantitative real time PCR (qPCR) is one of the most specific, sensitive and fast. Due to the small size of miRNA, a new qPCR method was developed to provide better specificity and sensitivity. This method includes two steps: stem-loop reverse transcription (RT) and qPCR [15]. In the first step, a stem-loop RT primer is hybridized to a miRNA molecule and pulsed RT is initiated. In the second step, the RT product is amplified with a specific forward primer and a universal reverse primer [15], [16]. RT and qPCR efficiencies are subjected to variation due to several factors including the amount and quality of starting materials. To take into account these variations, normalization is performed using endogenous control genes. It has been reported that it is better to normalize target RNA levels with control genes belonging to the same RNA class [17]. Therefore, for normalization of miRNA levels, the endogenous control genes would belong to the small non-coding RNA family (ncRNA), such as snRNA (small nuclear RNA) and snoRNA (small nucleolar RNA). Several candidate snRNAs and snoRNAs were tested across different tissues and experimental conditions to determine suitable endogenous controls [18]–[20]. However, no such analysis has yet been performed in the changing developing mouse lung.

snoRNAs are constitutively and abundantly expressed molecules found within the nucleolus where they are involved in chemical modification of various classes of RNAs [21]–[23]. For this study, we selected five snoRNAs that were already tested across different adult mouse tissues for stability [20]. We already demonstrated that the expression profile of many genes varies according to sex and developmental stage [24]. Expression of several miRNAs was also shown to vary according to sex and developmental age in the developing lung [25].

*Let-7* is highly conserved across animal species [26]. Some studies report the important role of *Let-7* in the development of *Caenorhabditis elegans*, *Drosophila*, and some mammals [27]–[30]. *Let-7* genes are expressed in the mouse developing lung [31] and are among the highly expressed miRNAs in the adult mouse lung [32]. They were shown to exert anti- and pro-inflammatory actions in respiratory diseases [32], [33], and to act as tumor suppressor in lung cancer [34]–[36].

In order to find endogenous control genes to study miRNA expression in the developing mouse lung of both sexes, expression stability of five putative snoRNA endogenous control genes was studied. The results are presented here. Calculation was performed separately for each developmental stage, and with all the time points together. The importance to select the appropriate combination of control genes is shown by qPCR relative quantification of *Let-7a* expression.

## Materials and Methods

### Animals and housing

Protocols were approved by the Comité de protection des animaux du CHU de Québec (protocol no. 2011-053). All animals were kept under a 12 h light/dark cycle and received water and feed ad libitum. Females and males Balb/c mice (Charles River Laboratories, St-Constant, Qc, Canada) were mated during a one hour window, which corresponded to gestational day (GD) 0.0. For animals sacrificed after birth, the beginning of postnatal day (PN) 0 corresponded to parturition. Pregnant females were sacrificed by exposure to CO_2_ and pups by decapitation following hypothermia-induced anesthesia (PN 0 to PN 5), or by intra-peritoneal injection of Euthanyl (PN 5 to PN 30). Fetal/neonatal lungs and hind legs were collected and snap frozen.

### Fetal sex determination

Fetal sex was determined by PCR amplification of the male-specific *Sry* gene (GenBank no. X67204) from fetal legs. DNA was extracted with Extracta DNA Prep for PCR – Tissue (Quanta BioSciences) as described by the manufacturer. PCR amplification was performed using AccuStart PCR SuperMix Kit (Quanta BioSciences) with 0.04 nM of each Sry primer (forward: 5′TATGGTGTGGTC CCGTGGTG-3′; reverse: 5′-ATGTGATGGCATGTGGGTTCC-3′), resulting in an amplicon of 282 nucleotides. The following PCR conditions were used: 94°C for 5 min and 72°C for 10 min followed by 34 cycles of 94°C for 1 min, 65°C for 1 min and 72°C for 1 min. Final extension was done at 72°C for 10 min. Agarose gel electrophoresis was used for amplicon visualization. Sex of neonates was determined by examination of the ano-genital distance and gonadal morphology.

### RNA isolation

For each litter, the whole lungs of fetal/neonatal mice were pooled by sex prior to homogenization. Three litters were pooled to create each biological replicate (Table 1). Total RNA was extracted using Tri-reagent, a mixture of phenol and guanidine thiocyanate in a monophasic solution (Molecular Research Center, Cincinnati, OH, USA), then purified on a CsCl gradient as previously described [37]. RNA integrity was verified using an Agilent 2100 Bioanalyzer (Agilent, Santa Clara, CA, U.S.A.). RIN values were between 7.5 and 10 for all the samples but two, which gave values of 6.3 and 6.4. RNA purity was determined using a Nanodrop 1000 spectrophotometer (Thermo Scientific). For all the samples, the OD260/280 ratio was over 1.95.

**Table 1:** Lung developmental stages and number of fetuses used.

Developmental stage	Age	Number of male fetuses/pool (n = 3 litters)	Number of female fetuses/pool (n = 3 litters)
Pseudoglandular	GD 15.5	2/2/4	6/3/2
Canalicular	GD 17.0	4/3/3	3/7/3
Saccular	GD 18.0	2/7/2	7/2/3
	PN 0	6/2/3	2/5/4
Alveolar	PN 7	3/5/3	2/2/2
	PN 15	3/2/2	3/2/3
	PN 30	2/2/3	2/2/4

GD, gestational day.

PN, postnatal day.

### Reverse transcription and quantitative PCR

RT and qPCR were performed as described by Varkonyi-Gasic and Hellens [16]. This method combines the advantages of using stem-loop RT primers specific to each analyzed miRNA and a pulsed RT reaction, two parameters that increase the specificity and sensitivity of detection. Briefly, 300 ng of each RNA template were denatured and mixed with 62.5 µM of each dNTP and 50 nM of the stem-loop primer at 65°C for 5 min, and then transferred on ice. First-strand buffer (SuperScript II kit, Life Technologies), 4 units of Protector RNase Inhibitor (Promega) and 50 units of SuperScript II RT (Life Technologies) were added to the mixture for a total reaction volume of 20 µl. Samples were incubated for 30 min at 16°C, followed by pulsed RT of 60 cycles at 30°C for 30 sec, 42°C for 30 sec and 50°C for 1 sec. Reverse transcriptase was then inactivated for 5 min at 85°C. No-template control and no-reverse transcriptase control were performed and no amplicon was detected. qPCR was performed using the LightCycler FastStart DNA Master SYBR Green I kit (Roche Diagnostics) and a Light Cycler device (Roche Diagnostics). Reactions were performed according to the manufacturer's instructions with 0.5 µM of each primer (final concentration) and 30 ng of total RNA input in a final volume of 20 µl. Samples were incubated at 95°C for 5 min, followed by 43 cycles of 95°C for 5 sec and 60°C for 10 sec. At the end of each run, samples were heated to 95°C with a temperature transition rate of 0.2°C/sec to construct dissociation curves. Amplicons from all the amplified genes were sequenced showing the specificity of PCR reactions. Primers used in this study are listed in Table 2. A technical duplicate was performed for each biological replicate.

**Table 2:** Putative endogenous control genes and primers used for reverse transcription and qPCR.

Gene	NCBI Accession Number	Reverse transcription primer^a^^,^ b	PCR Efficiency
		qPCR forward primer^c^^,^ d	
**sno135**	AF357323	**RT** 5′-GTTGGCTCTGGTGCAGGGTCCGAGGTATTCGCACCAGAGCCAAC**CTTCAG**-3′	1,83
		**F** 5′-GCGGCGGCTAAAATAGCTGGAA-3′	
**sno142**	AF357324	**RT** 5′-GTTGGCTCTGGTGCAGGGTCCGAGGTATTCGCACCAGAGCCAAC**TTCCTC**-3′	1,99
		**F** 5′-GCGGCGGGTCAGTGCCACGTGT-3′	
**sno202**	AF357327	**RT** 5′-GTTGGCTCTGGTGCAGGGTCCGAGGTATTCGCACCAGAGCCAAC**CATCAG**-3′	1,95
		**F** 5′-GCGGCGGGCTGTACTGACTTGA-3′	
**sno234**	AF357329	**RT** 5′-GTTGGCTCTGGTGCAGGGTCCGAGGTATTCGCACCAGAGCCAAC**TCTCAG**-3′	1,81
		**F** 5′-GCGGCGGCTTTTGGAACTGAAT-3′	
**sno251**	AF357332	**RT** 5′-GTTGGCTCTGGTGCAGGGTCCGAGGTATTCGCACCAGAGCCAAC**CTGGCT**-3′	1,99
		**F** 5′-GCGGCGGATACATACTTGCCCT-3′	

^*a*^Nucleotide sequences in bold are specific to each gene.

^*b*^RT: Reverse transcription.

^*c*^The reverse primer for qPCR is the same for all the genes and corresponds to a segment of the reverse transcription primers: 5′-GTGCAGGGTCCGAGGT-3′ [16].

^*d*^F: Forward.

### Housekeeping gene expression stability and data analysis

Stability of housekeeping genes was assessed with three different programs: geNorm, NormFinder, and BestKeeper. geNorm (v.3.4) calculates the gene expression stability measure *M* for a control gene as the average pairwise variation *V* for that gene with all other studied control genes [17]. Stepwise exclusion of the gene with the highest *M* value allows ranking of the tested genes according to their expression stability [17]. The geNorm applet calculates also one gene expression normalization factor for each tissue sample based on the geometric mean of the selected reference genes [17]. NormFinder (v.20) is an applet identifying the optimal normalisation gene(s) among a set of candidate genes. It uses an ANOVA-based model to estimate intra- and inter-group variations, and it ranks the set of candidate normalization genes conforming to their expression stability [38]. Bestkeeper (v1.0) determine the expression stability of control genes from the Cq values by calculating standard deviation, percentage of covariance and coefficient of correlation. A BestKeeper Index is calculated for each sample as the geometric mean of Cq values of control genes, and the correlation between each candidate gene and the index is calculated to obtain the coefficient of correlation [39].

For relative quantification of Let-7a RNA levels, the standard curves required for the external standard normalization method were prepared as previously described [40]. Normalization factors were calculated with geNorm as described above.

### Statistics

Statistical analyses were performed using GraphPad Prism 5.01 (GraphPad Software, La Jolla, CA, USA). A paired Student t-test was used to compare the expression values of *Let7-a* between sexes. A p-value ≤0.05 was considered to be significant.

## Results and Discussion

### Expression stability of putative endogenous control genes

Selection of inappropriate control genes can introduce pseudo-variations or hide real biological variations. Because the developing lung is changing over time, quantification of miRNA expression requires careful selection of endogenous control genes according to the studied period of development. Because some developmental events are delayed in male lungs compared with female lungs [37], [41]–[44], the sex has also to be considered. The selected samples (Table 1) covered four developmental stages extending from the end of the pseudoglandular stage (gestation day (GD) 15) to the end of the alveolar stage (postnatal day (PN) 30). This developmental period includes lung maturation and alveolarization, which are respectively related to respiratory distress syndrome and bronchopulmonary dysplasia, two major diseases frequently observed in cases of preterm birth. One pool per sex per litter and three litters per time point were analyzed. Because the use of multiple control genes is highly recommended for normalization of RT-qPCR data [17], five putative endogenous snoRNA control genes were selected (Table 2). These snoRNAs were subjected to a non-exhaustive expression study with adult mouse tissues by Wong et al. [20] and *sno202* was proposed as normalization gene because it showed the highest abundance and least variability across the 12 tested tissues.

**Figure 1:**
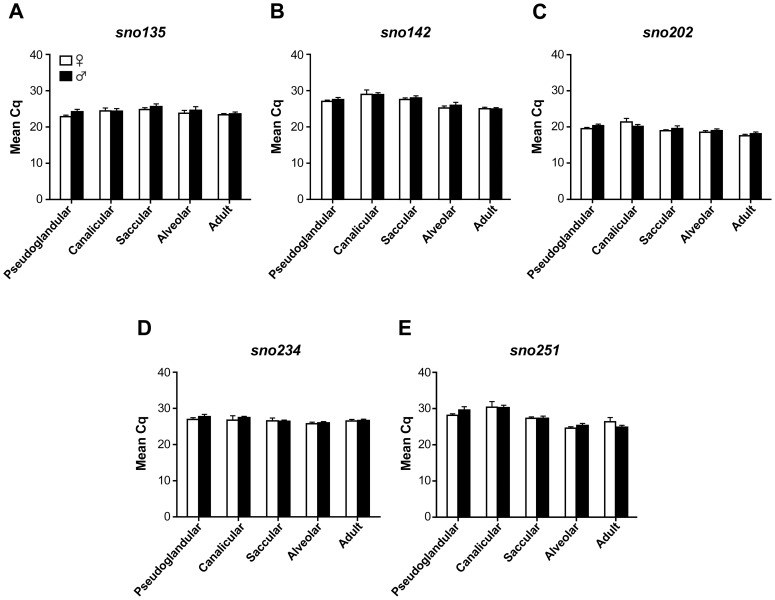
Expression levels of putative housekeeping genes in the mouse lung at different developmental stages and in the adult lung. Cq (mean ± SEM) obtained by qPCR are presented for the five putative control genes for the indicated developmental stages and for adult lungs. Pools of male and female lungs were used (see Table 1 for details).

In this study, RT-qPCR was performed to quantify expression of *sno135*, *sno142*, *sno202*, *sno234*, and *sno251*. The results were expressed as mean Cq (quantification cycle) (Figs. 1 and S1), which is the standard name for Ct or Cp according to the Real-time PCR Data Markup Language (RDML) guidelines [45]. The gene to gene differences between the Cq values were quite similar for all the tested developmental time points (Fig. S1). The most expressed gene was *sno202* for both sexes at all the tested developmental stages, which is consistent with the study of Wong *et al*. performed on adult mouse tissues, including the lung [20]. *sno251* showed the higher variation across the different developmental stages, while Cq values of *sno234* were the most stable from stage to stage (Fig. 1).

Several softwares were developed to analyze the expression stability of reference genes, the most largely used being geNorm, NormFinder and BestKeeper. They are used here. geNorm calculates the stability value *M* based on the arithmetic mean of all pairwise variations to determine the stability of control genes; the lower the *M* value, the higher the stability [17]. NormFinder estimates the overall expression variation of the candidate normalization genes, as well as the intra-group and the inter-group variations [38]. Again, decreasing stability values indicate increasing gene expression stability. The two programs determine also the best pair from a panel of control genes. geNorm proceeds by stepwise exclusion of the gene with the highest *M* value, and a new *M* value is calculated for the remaining genes, ending with a combination of the two most stable genes. The ranking of genes vary during this process. geNorm also provides the optimal number of reference genes required for normalization. NormFinder selects two best genes with minimal combined inter- and intra- group expression variation. Generally, the results from both softwares are slightly different but consistent. BestKeeper was used to generate SD values: the lower the SD value, the higher the gene stability.

We assessed the stability of putative control genes in the developing lung at specific developmental stages to determine the most stable genes based on stability values calculated by geNorm, NormFinder and BestKeeper. The stability values were determined for each developmental stage separately for each sex (Table 3) and with both sexes combined (Table 4). Our data demonstrate that the relative gene stability may vary throughout the developmental period. For example, in males, *sno142* ranked first during the saccular stage while it was the least stable gene during the canalicular stage with geNorm and NormFinder (Table 3). *sno234* was the least stable gene during the saccular stage and the most stable gene during the pseudoglandular and the alveolar stages with both geNorm and NormFinder (Table 4). In addition, *sno251* was the least stable gene during the pseudoglandular and the canalicular stages with the three calculation methods, while it was the second more stable gene during the alveolar stage with geNorm and NormFinder (Table 4). These observations are compatible with the fact that the developing lung is changing across developmental time.

**Table 3:** Stability values of housekeeping gene expression in lungs at different developmental stages for each sex.

Developmental Stage	Sex	Rank	geNorm^a^		NormFinder^b^		BestKeeper^c^	
			Gene	Stability	Gene	Stability	Gene	SD
Pseudoglandular	**♀**	1	*sno234*	0,186	*sno234*	0,049	*sno202*	0,248
		2	*sno142*	0,212	*sno142*	0,085	*sno142*	0,313
		3	*sno202*	0,258	*sno251*	0,149	*sno234*	0,370
		4	*sno251*	0,265	*sno202*	0,156	*sno251*	0,488
		5	*sno135*	0,292	*sno135*	0,174	*sno135*	0,649
	**♂**	1	*sno234*	0,263	*sno234*	0,026	*sno202*	0,588
		2	*sno142*	0,289	*sno142*	0,096	*sno135*	0,778
		3	*sno135*	0,313	*sno135*	0,174	*sno234*	0,862
		4	*sno202*	0,328	*sno202*	0,192	*sno142*	0,891
		5	*sno251*	0,463	*sno251*	0,300	*sno251*	1,141
Canalicular	**♀**	1	*sno142*	1,289	*sno142*	0,390	*sno135*	1,054
		2	*sno135*	1,344	*sno135*	0,477	*sno142*	1,401
		3	*sno234*	1,609	*sno202*	0,919	*sno202*	1,433
		4	*sno202*	1,614	*sno234*	1,005	*sno234*	1,473
		5	*sno251*	1,784	*sno251*	1,144	*sno251*	2,069
	**♂**	1	*sno135*	0,572	*sno135*	0,170	*sno234*	0,424
		2	*sno202*	0,591	*sno234*	0,312	*sno202*	0,506
		3	*sno234*	0,605	*sno202*	0,314	*sno142*	0,701
		4	*sno251*	0,778	*sno251*	0,434	*sno135*	0,792
		5	*sno142*	0,843	*sno142*	0,510	*sno251*	0,977
Saccular	**♀**	1	*sno251*	0,900	*sno251*	0,271	*sno202*	0,351
		2	*sno142*	0,936	*sno142*	0,383	*sno142*	0,555
		3	*sno135*	0,975	*sno135*	0,454	*sno251*	0,785
		4	*sno202*	1,025	*sno202*	0,480	*sno135*	0,942
		5	*sno234*	1,392	*sno234*	0,878	*sno234*	1,226
	**♂**	1	*sno142*	1,141	*sno142*	0,188	*sno234*	0,433
		2	*sno234*	1,383	*sno234*	0,688	*sno142*	0,857
		3	*sno202*	1,478	*sno202*	0,796	*sno251*	1,096
		4	*sno135*	1,495	*sno135*	0,824	*sno202*	1,356
		5	*sno251*	1,525	*sno251*	0,880	*sno135*	1,391
Alveolar	**♀**	1	*sno234*	1,650	*sno234*	0,127	*sno234*	0,725
		2	*sno142*	1,894	*sno251*	0,699	*sno251*	1,084
		3	*sno251*	1,975	*sno142*	0,756	*sno142*	1,252
		4	*sno135*	2,149	*sno135*	1,256	*sno202*	1,277
		5	*sno202*	2,883	*sno202*	1,877	*sno135*	2,098
	**♂**	1	*sno234*	2,332	*sno251*	0,652	*sno202*	0,841
		2	*sno251*	2,359	*sno234*	0,838	*sno234*	0,857
		3	*sno142*	2,774	*sno142*	1,341	*sno251*	1,298
		4	*sno135*	2,781	*sno135*	1,610	*sno142*	1,509
		5	*sno202*	3,293	*sno202*	2,014	*sno135*	2,424
Developing lung^d^	**♀**	1	*sno234*	1,697	*sno234*	0,481	*sno234*	0,994
		2	*sno142*	1,873	*sno142*	0,819	*sno202*	1,070
		3	*sno135*	2,010	*sno135*	1,034	*sno135*	1,445
		4	*sno251*	2,109	*sno251*	1,069	*sno142*	1,482
		5	*sno202*	2,444	*sno202*	1,456	*sno251*	2,008
	**♂**	1	*sno234*	1,939	*sno234*	0,604	*sno234*	0,912
		2	*sno142*	2,243	*sno142*	1,052	*sno202*	1,074
		3	*sno251*	2,272	*sno251*	1,115	*sno142*	1,573
		4	*sno135*	2,363	*sno135*	1,258	*sno135*	1,697
		5	*sno202*	2,572	*sno202*	1,461	*sno251*	1,987
Adult	**♀**	1	*sno234*	0,515	*sno142*	0,017	*sno135*	0,178
		2	*sno142*	0,527	*sno234*	0,017	*sno234*	0,303
		3	*sno135*	0,560	*sno135*	0,063	*sno142*	0,328
		4	*sno202*	1,132	*sno202*	0,767	*sno202*	0,465
		5	*sno251*	1,156	*sno251*	0,785	*sno251*	1,138
	**♂**	1	*sno135*	0,309	*sno135*	0,048	*sno142*	0,168
		2	*sno202*	0,343	*sno142*	0,061	*sno234*	0,205
		3	*sno142*	0,352	*sno202*	0,145	*sno135*	0,368
		4	*sno251*	0,429	*sno251*	0,271	*sno202*	0,420
		5	*sno234*	0,724	*sno234*	0,498	*sno251*	0,577

^*a*^ *M* stability values are calculated by geNorm. Gene stability increases while *M* value decreases.

^*b*^Stability values are calculated by NormFinder, Gene stability increases while stability value decreases.

^*c*^Standard deviation (SD) is calculated by BestKeeper. Gene stability increases while SD value decreases.

^*d*^All studied developmental stages.

**Table 4:** Housekeeping gene stability values in lungs at different developmental stages with both sexes combined.

Stage	Rank	geNorm^a^		NormFinder^b^		BestKeeper^c^	
		Gene	Stability	Gene	Stability	Gene	SD
Pseudoglandular	1	*sno234*	0,259	*sno234*	0,073	*sno202*	0,468
	2	*sno142*	0,299	*sno142*	0,134	*sno142*	0,612
	3	*sno202*	0,326	*sno202*	0,144	*sno234*	0,694
	4	*sno135*	0,342	*sno135*	0,146	*sno135*	0,849
	5	*sno251*	0,437	*sno251*	0,195	*sno251*	1,058
	**Best combination**	*sno142/sno234*	0,130	*sno135/sno234*	0,074		
Canalicular	1	*sno135*	1,013	*sno135*	0,187	*sno135*	0,923
	2	*sno142*	1,068	*sno142*	0,260	*sno142*	1,023
	3	*sno234*	1,238	*sno202*	0,356	*sno234*	1,037
	4	*sno202*	1,336	*sno234*	0,380	*sno202*	1,124
	5	*sno251*	1,341	*sno251*	0,456	*sno251*	1,550
	**Best combination**	*sno135*/*sno234*	0,601	*sno135/sno142*	0,166		
Saccular	1	*sno142*	1,027	*sno142*	0,116	*sno142*	0,697
	2	*sno135*	1,244	*sno251*	0,235	*sno234*	0,830
	3	*sno202*	1,244	*sno202*	0,261	*sno202*	0,909
	4	*sno251*	1,260	*sno135*	0,261	*sno251*	0,938
	5	*sno234*	1,404	*sno234*	0,320	*sno135*	1,299
	**Best combination**	*sno135*/*sno142*	0,752	*sno142/sno251*	0,139		
Alveolar	1	*sno234*	1,990	*sno234*	0,161	*sno234*	0,780
	2	*sno251*	2,139	*sno251*	0,225	*sno202*	1,066
	3	*sno142*	2,351	*sno142*	0,350	*sno251*	1,262
	4	*sno135*	2,445	*sno135*	0,478	*sno142*	1,423
	5	*sno202*	3,023	*sno202*	0,649	*sno135*	2,237
	**Best combination**	*sno135*/*sno234*	0,147	*sno234/sno251*	0,222		
Developing lung^d^	1	*sno234*	1,816	*sno234*	0,118	*sno135*	1,586
	2	*sno142*	2,058	*sno142*	0,204	*sno142*	1,527
	3	*sno251*	2,178	*sno251*	0,238	*sno234*	0,955
	4	*sno135*	2,179	*sno135*	0,250	*sno202*	1,069
	5	*sno202*	2,488	*sno202*	0,318	*sno251*	1,979
	**Best combination**	*sno135*/*sno234*	1,466	*sno142/sno234*	0,118		
Adult	1	*sno142*	0,528	*sno142*	0,044	*sno142*	0,248
	2	*sno135*	0,538	*sno135*	0,134	*sno234*	0,254
	3	*sno234*	0,643	*sno234*	0,255	*sno135*	0,273
	4	*sno202*	0,867	*sno202*	0,452	*sno202*	0,443
	5	*sno251*	1,110	*sno251*	0,521	*sno251*	0,969
	**Best combination**	*sno135*/*sno142*	0,082	*sno135/sno142*	0,083		

^*a*^ *M* stability values calculated with the geNorm software.

^*b*^Stability values calculated with the NormFinder software.

^*c*^Standard deviation (SD) is calculated by BestKeeper. Gene stability increases while SD value decreases.

^*d*^All studied developmental stages.

Ranking of genes according to expression stability can also vary between sexes. For example, during the canalicular stage, *sno142* was the most stable gene in females and the least stable gene in males by geNorm and NormFinder (Table 3). During the saccular stage, the same situation occurred with *sno251*. In contrast, BestKeeper did not show these sex differences. However, for *sno234*, the three calculation methods showed a sex difference in ranking during the saccular stage (Table 3) with less stability in females. The SD values for BestKeeper were 1.226 for females and 0.433 for males. In adult lungs, the most stable gene by geNorm and NormFinder was *sno234* in females but not in males where it was the least stable gene (Table 3). Taken together, our data show that gestation time and sex may both influence gene stability and ranking.

**Figure 2:**
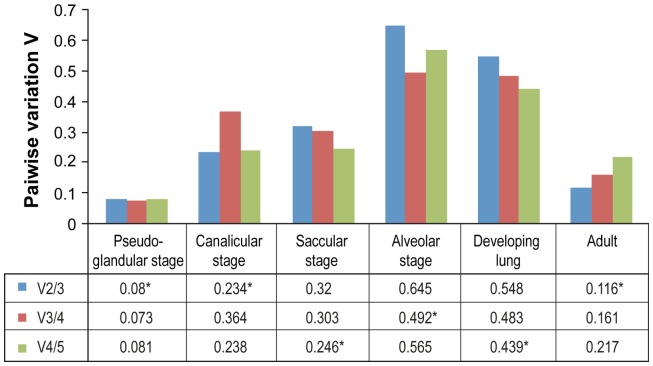
Determination of optimal number of reference genes for normalization. The pairwise variation (Vn/Vn+1) was analyzed between two sequential normalization factors by geNorm. The optimal number of reference genes varies according to the developmental stage: two genes for the pseudoglandular and the canalicular stages as well as for the adult, three genes for the alveolar stage, and four genes for the saccular stage and the developing lung (from GD 15.5 to PN 30) as indicated by an asterisk.

The optimal number of reference genes for normalization was calculated by geNorm for each developmental stage for both sexes combined (Fig. 2). The optimal number of genes varied from 2 to 4 according to the period of gestation studied. The optimal number of genes is recommended for the study of small expression differences. We have compared the use of the best pair of reference genes *vs* the optimal number of reference genes as proposed by geNorm for the analysis of the sex difference in *Let7a* expression during the saccular and the alveolar stages. No statistically significant sex difference was obtained at all (saccular stage: P = 0.2188 and 0.2857 for 4 and 2 reference genes, respectively; alveolar stage: P = 0.8203 and 0.9102 for 3 and 2 genes, respectively). Based on our data and on previous reports [18], , we propose using the best pairs of reference genes.

Best pair combination of normalization genes were calculated using samples of both sexes for each developmental stage (Table 4). In fact, the two more stable genes were not necessarily the best pair combination, which can be determined by geNorm and NormFinder based on the variability of a pair of genes instead of one gene. The pairs of genes proposed by geNorm and NormFinder were identical only for the adult lungs (*sno135*/*sno142*), which were in fact the most homogenous materials, in contrast to developing lungs which were not necessarily perfectly synchronized. However, at least one proposed gene of each pair was identical between the two methods for each developmental stage and all the proposed pairs had acceptable stability values. By geNorm, the best pair combinations were *sno142*/*sno234* for the pseudoglandular stage, *sno135*/*sno234* for the canalicular and the alveolar stages, and *sno135*/*sno142* for the saccular stage. In NormFinder, *sno135*/*sno234*, *sno135*/*sno142*, *sno142*/*sno251*, and *sno234*/*sno251* were the best gene combinations for the pseudoglandular, canalicular, saccular and the alveolar stages, respectively.

We have also assessed the stability of control genes with all developmental stages and both sexes combined to determine which pairs of control genes were the most stable across lung development (Table 4). In geNorm, the best pair combination was *sno135*/*sno234*, while *sno142*/*sno234* was proposed by NormFinder.

As shown above, the five genes were not systematically ranked in the same order by geNorm, NormFinder and BestKeeper. Such differences between the three methods are expected and were already reported [50]–[52] because the three algorithms use different mathematical models. In addition we observed that geNorm and NormFinder generated more similar ranking compared with BestKeeper (Table 3 and 4). Such a phenomenon was also reported by Jiang et al. [51]. This can be explained by the fact that, in contrast to geNorm and NormFinder, BestKeeper estimates the variation of each single gene independently. Nevertheless, some differences in gene ranking were also observed between geNorm and NormFinder (Table 3 and 4), as already reported [50]–[53].

### Effect of endogenous control genes on the measure of relative Let7-a expression level

**Figure 3:**
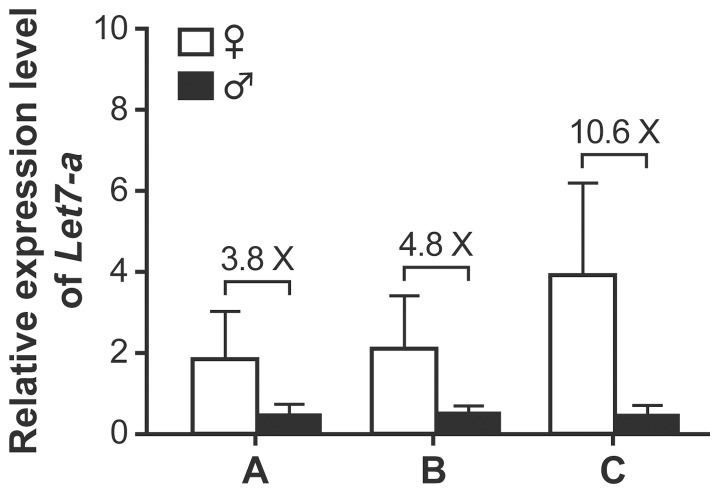
Relative expression levels of *Let-7a* in canalicular-stage lungs normalized with different pairs of housekeeping genes. Let7-a RNA levels obtained by qPCR were normalized using the best pair of control genes calculated with: A) geNorm (*sno135*/*sno234*); B) NormFinder (*sno135*/*sno142*). C) qPCR data were normalized using a pair of less stable genes as estimated by geNorm and NormFinder (*sno202*/*sno251*). Pools of male and female lungs were used (see Table 1 for details).

**Figure 4:**
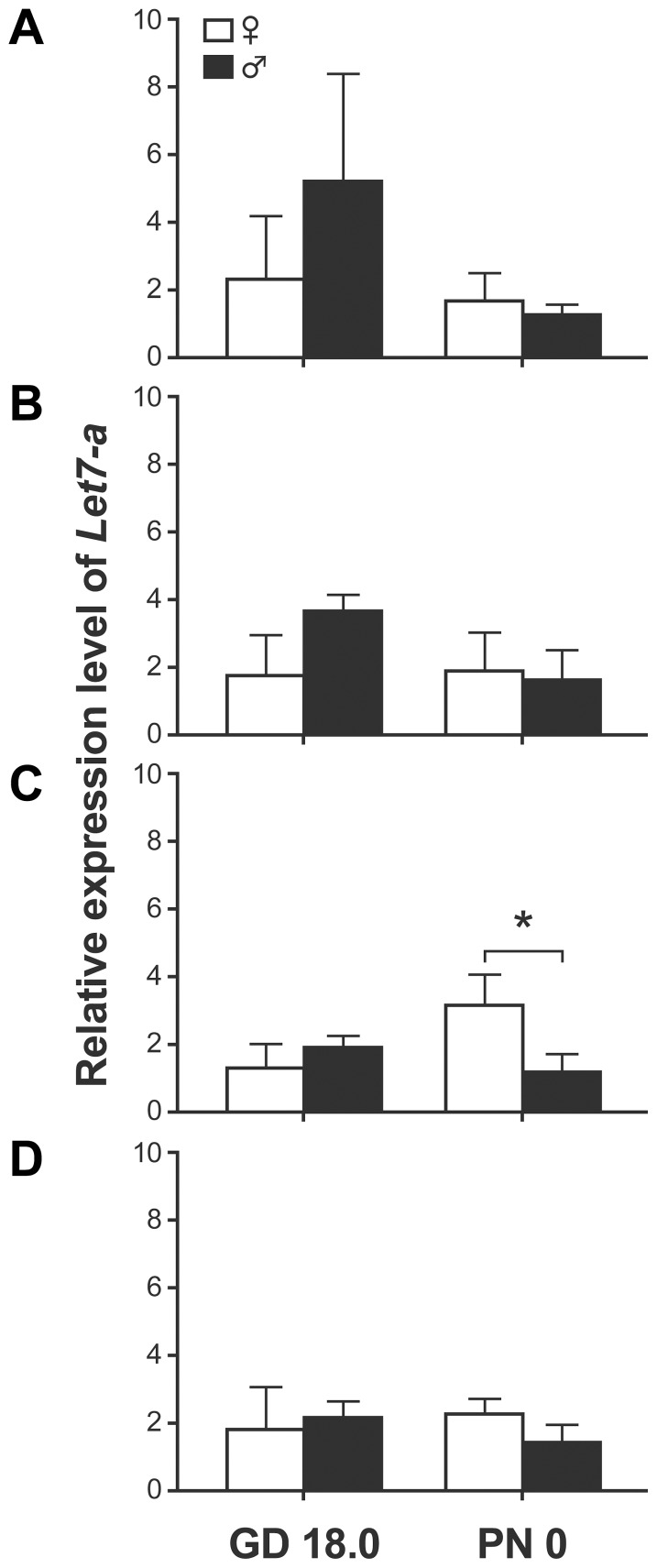
Relative expression levels of *Let-7a* in saccular-stage lungs normalized with different pairs of housekeeping genes. Let7-a RNA levels obtained by qPCR are presented for lung RNA samples collected during the saccular stage on GD 18.0 and PN 0. Data were normalized using the best pair of control genes calculated with the saccular stage samples and with: A) geNorm (*sno135*/*sno142*); B) NormFinder (*sno142*/*sno251*). In (C), a pair of less stable genes as estimated either by geNorm or NormFinder was used (*sno202*/*sno234*) and a significant sex difference was observed on PN 0 (*, P = 0.018, Student t-test). When the best pair calculated by geNorm using all the samples from pseudoglandular to alveolar stages (*sno135*/*sno234*) was used (D), no significant sex difference was observed on PN 0 (P = 0.109, Student t-test). Pools of male and female lungs were used (see Table 1 for details).

To determine the effect of the selection of normalization genes on quantification of miRNA expression, we quantified Let-7a RNA levels using different pairs of control genes. First, we selected the best pairs of genes sorted by geNorm and NormFinder for the canalicular stage and compared the results with those obtained with another pair of genes. The magnitude of the sex difference in *let7-a* expression levels evaluated with the two proposed pairs of genes was similar (3.8× vs 4.8×), whereas the data normalized with the least stable pair of genes showed higher sex differences (10.6×) (Fig. 3). Second, we reproduced the same experiment but using samples from GD 18.0 and PN 0 of the saccular stage separately. No statistically significant sex difference in *let7-a* expression levels was observed on GD 18.0 with either the best pairs of normalization genes calculated with all the samples of the saccular stage by geNorm (*sno135*/*sno142*) and NormFinder (*sno142*/*sno251*), or the least stable control genes (*sno234*/*sno202*) (Fig. 4). However, for males on GD 18.0, a higher variability between biological replicates was observed with the best control genes selected by geNorm compared with those selected by NormFinder. For an unknown reason, such a higher variability was not observed for all the experimental conditions normalized with this pair of control genes (*sno135*/*sno142*). With samples from PN 0, no sex difference was observed in *let7-a* expression levels using the best pair of control genes from the two calculation methods, while a statistically significant sex difference (p<0.02) was observed with the other pair. Therefore, the use of the two calculation methods led to similar conclusions, in contrast to the use of the less stable pair of normalization genes. We also used the most stable pair of genes calculated by geNorm with data from the entire studied developmental window (*sno135*/*sno234*). When normalized with these control genes, the *let7-a* expression data showed no statistically significant sex difference, which is the same conclusion as with the best stable control genes calculated with geNorm and NormFinder using only the samples from the saccular stage (Fig. 4D). The fact that the developing lung is changing implies that normalization genes should be selected within the analyzed time window. The use of control genes selected from multi-stage sampling would be reserved for studies extending over multiple developmental stages. Taken together, our data demonstrate the importance of choosing the most stable pair of endogenous control genes to adequately represent the actual biological situation.

## Conclusion

Recent studies quantifying miRNAs by qPCR in the developing lung used normalization genes known to be stable in various adult tissues [54]–[56]. To our knowledge no study has focused on analyzing the expression stability of control genes in the lung by sex and over developmental time. Our study analyzes the stability expression of five endogenous control genes through lung development and by sex. Our data demonstrate that ranking of genes according to expression stability is influenced by sex and developmental age when geNorm, NormFinder or BestKeeper is used. We present for the first time pairs of control genes for specific developmental stages as well as for the entire period extending from the pseudoglandular to the alveolar stages of lung development, which corresponds to the most studied period. These findings will be helpful for studies of miRNA involvement in lung development and neonatal diseases related to preterm birth.

## Supporting Information

Figure S1 **Comparison of expression levels of putative housekeeping genes in the mouse developing lung and the adult lung.** Cq (mean ± SEM) obtained by qPCR are presented for the five putative control genes for the indicated developmental stages and for adult lungs. Pools of male and female lungs were used (see Table 1 for details). The data are the same than in Fig. 1 but are presented differently.
(TIF)
