# Supplementary figures and images for: Identification of Most Stable Endogenous Control Genes for MicroRNA Quantification in the Developing Mouse Lung
Source: PLoS One. 2014 Nov 4;9(11):e111855. doi: 10.1371/journal.pone.0111855 (PMC4219792; doi:10.1371/journal.pone.0111855)

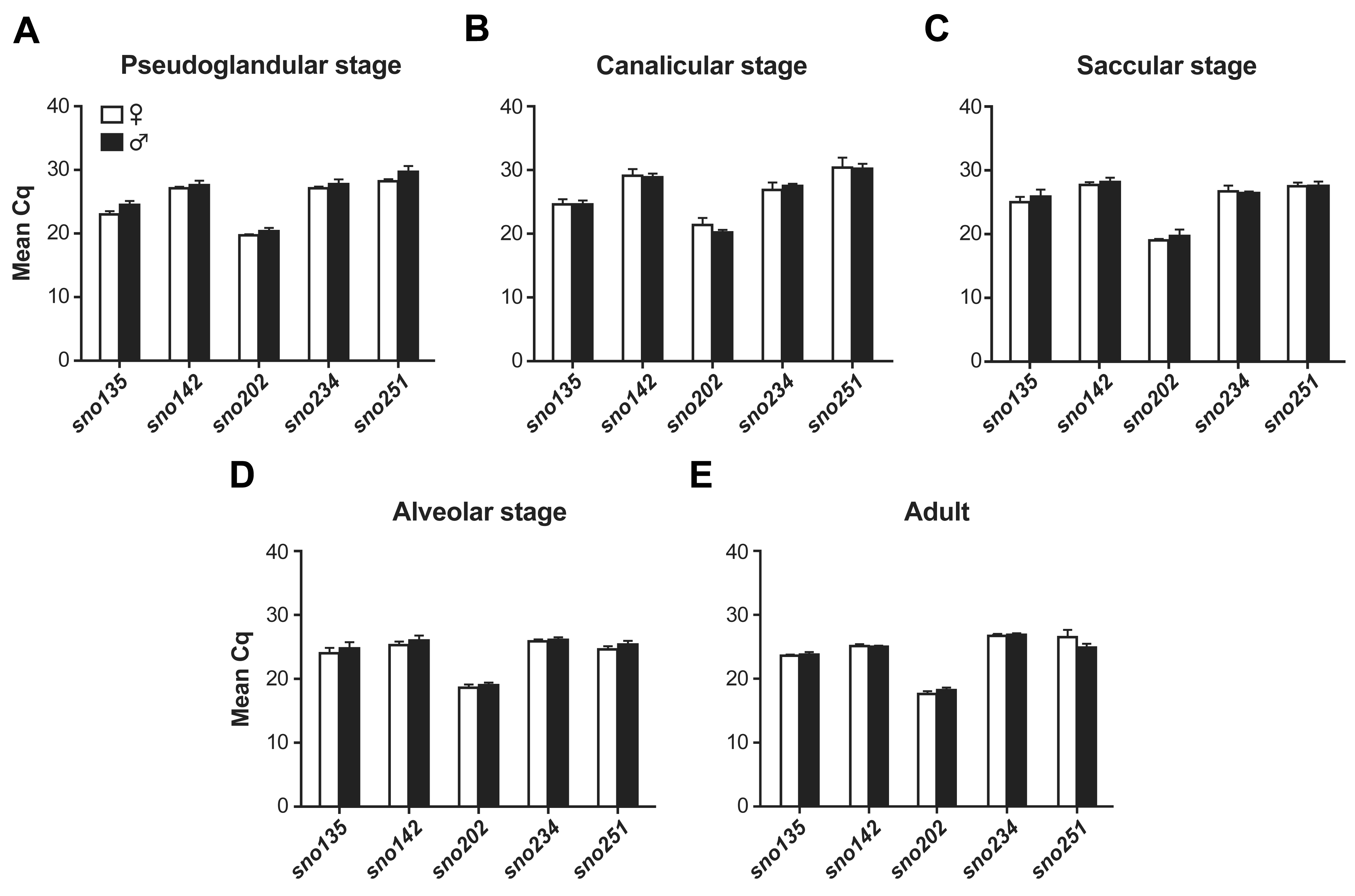

Supplement: Figure S1 — Comparison of expression levels of putative housekeeping genes in the mouse developing lung and the adult lung. Cq (mean ± SEM) obtained by qPCR are presented for the five putative control genes for the indicated developmental stages and for adult lungs. Pools of male and female lungs were used (see Table 1 for details). The data are the same than in Fig. 1 but are presented differently. (TIF) [file pone.0111855.s001.tif]
